# Magnesium Picolinate Improves Bone Formation by Regulation of RANK/RANKL/OPG and BMP-2/Runx2 Signaling Pathways in High-Fat Fed Rats

**DOI:** 10.3390/nu13103353

**Published:** 2021-09-24

**Authors:** Emre Sahin, Cemal Orhan, Tansel Ansal Balci, Fusun Erten, Kazim Sahin

**Affiliations:** 1 Department of Animal Nutrition, Faculty of Veterinary Medicine, Firat University, Elazig 23119, Turkey; esahin@bingol.edu.tr (E.S.); corhan@firat.edu.tr (C.O.); 2 Department of Nuclear Medicine, School of Medicine, Firat University, Elazig 23119, Turkey; tanselbalci@firat.edu.tr; 3 Department of Veterinary Medicine, Pertek Sakine Genc Vocational School, Munzur University, Tunceli 62500, Turkey; fusunerten@munzur.edu.tr

**Keywords:** magnesium picolinate, bone, bone mineral density, osteogenic proteins, high-fat diet

## Abstract

Magnesium (Mg) deficiency may affect bone metabolism by increasing osteoclasts, decreasing osteoblasts, promoting inflammation/oxidative stress, and result in subsequent bone loss. The objective of the present study was to identify the molecular mechanism underlying the bone protective effect of different forms of Mg (inorganic magnesium oxide (MgO) versus organic magnesium picolinate (MgPic) compound) in rats fed with a high-fat diet (HFD). Forty-two Wistar albino male rats were divided into six group (*n* = 7): (i) control, (ii) MgO, (iii) MgPic, (iv) HFD, (v) HFD + MgO, and (vi) HFD + MgPic. Bone mineral density (BMD) increased in the Mg supplemented groups, especially MgPic, as compared with the HFD group (*p <* 0.001). As compared with the HFD + MgO group, the HFD + MgPic group had higher bone P (*p* < 0.05) and Mg levels (*p <* 0.001). In addition, as compared to MgO, MgPic improved bone formation by increasing the levels of osteogenetic proteins (COL1A1 (*p <* 0.001), BMP2 (*p <* 0.001), Runx2 (*p <* 0.001), OPG (*p* < 0.05), and OCN (*p* < 0.001), IGF-1 (*p* < 0.001)), while prevented bone resorption by reducing the levels of RANK and RANKL (*p* < 0.001). In conclusion, the present data showed that the MgPic could increase osteogenic protein levels in bone more effectively than MgO, prevent bone loss, and contribute to bone formation in HFD rats.

## 1. Introduction

Magnesium (Mg) is one of the most abundant intracellular cations in the body, and it is involved in many metabolic events such as glycolysis, DNA synthesis and transcription, protein synthesis, cellular ion flow, cell membrane voltage control [1], and, in particular, bone metabolism [2]. It is mainly absorbed from the small intestines through the paracellular pathway, and bone stores nearly 60% of the total Mg content of the body. Serum Mg concentration and bone metabolism are related, and Mg is constantly exchanged between bone and blood [3]. Mg deficiency may trigger osteoclastic activity in bone by releasing inflammatory cytokines (tumor necrosis factor α (TNFα) and interleukin-1β (IL-1β)) and increasing the expression of osteoclast-related genes (in particular, receptor activator of nuclear factor-κB (RANK) and its activator RANK ligand (RANKL)] [2,4], whereas reducing osteoprotegerin (OPG), a decoy receptor of RANKL, levels in bone [5]. Thus, the RANK/RANKL/OPG pathway regulation could be impaired by Mg deficiency [6]. Similarly, inadequate Mg intake may increase inflammatory bone resorption and decrease bone mineral density (BMD) [7]. Mg supplements may maintain BMD by stimulating osteoblastogenesis and chondrogenesis while inhibiting inflammation-mediated osteoclastogenesis [3]. Mg increases osteoblastic bone formation by stimulating the expression of insulin-like growth factor 1 (IGF-1) in osteoblasts [8]. In addition, Mg increases the expression levels of BMP2 [9] and type 1 collagen in bone tissue [10].

The prevalence of obesity resulting from an unhealthy Western-type diet, high energy intake, or a sedimentary lifestyle has been increasing rapidly in humans [11] and animals [12]. Recent studies have shown that excessive high-fat diet (HFD) consumption and severe obesity adversely affect bone health [13,14,15] by impairing BMD through mechanical, hormonal, and inflammatory factors [14,16,17]. HFD intake increases the bone levels of TNF-α, IL-1β, and RANKL and attenuates the antioxidant capacity of bone [18]. Increased inflammatory factors suppress bone formation, leading to an increase in the RANKL/OPG ratio [18] because RANKL stimulates RANK in hematopoietic osteoclast progenitors and initiates osteoclastogenic differentiation [2]. Similarly, IGF-1 [18], osteocalcin (OCN) [19], bone morphogenetic protein 2 (BMP2), and type 1 collagen levels in the bone matrix could be reduced after HFD treatments in rodents [20]. Moreover, intestinal mineral absorption and mineral homeostasis in kidneys may be interrupted by HFD intake. Then, Ca and Mg metabolisms, which are essential for bone formation, may be disrupted [21,22].

Due to their higher solubility, most organic Mg forms are more bioavailable than inorganic Mg forms [23]. However, magnesium oxide (MgO) contains 60% elemental Mg, and the bioavailability of MgO is relatively lower than other Mg compounds due to weak solubility in water [24]. Magnesium picolinate (MgPic), synthesized from various Mg salts and picolinic acid, has a high solubility in the physiological pH range. Although MgPic contains 9% elemental Mg, it may show higher bioavailability than other Mg forms, such as MgO [25]. Previous studies have demonstrated that MgPic, rather than MgO, had more anti-inflammatory and antioxidant effects in the brain [26] and retina in rats [27]. The effects of different forms of magnesium (MgPic vs. MgO) on the RANK/RANKL/OPG and BMP-2/Runx2 signaling pathways associated with bone mineral density have not yet been fully investigated in rats fed with HFD. We hypothesized that MgPic, as compared with MgO, could accelerate bone formation under high-fat diet feeding in rats. Therefore, the objective of this study was to compare the protective effect of MgPic and MgO on bone formation by evaluating BMD and osteoclastogenic, osteogenic, and chondrogenic proteins.

## 2. Materials and Methods

### 2.1. Animals

Forty-two male Wistar albino rats (8 weeks and 180 ± 20 g) were used in the study. The total number of rats was determined by power analysis (0.05 type 1 error, 85% power, and 0.65 effect size) using the G*power program (Version 3.1.9.3, Heinrich-Heine-Universität Düsseldorf, Düsseldorf, Germany) [28,29]. Each group of rats (*n* = 7) were housed in two cages (polypropylene) as three or four animals, under a 12/12 h light/dark cycle, 22 °C, and 55% ± 5% humidity in the Experimental Research Center of Firat University (FUDAM). The study was approved by the Fırat University Animal Experiments Local Ethics Committee (08-2019/05). The standard (control) diet or high-fat diet (HFD) (Table 1) and water were provided ad libitum.

### 2.2. Experimental Design

After a two week acclimation period, the rats were randomly dived into six groups (*n* = 7) as follows: (i) control; rats fed a standard diet; (ii) MgO; rats fed a standard diet containing 500 mg/kg elemental Mg as MgO; (iii) MgPic; rats fed a standard diet containing 500 mg/kg elemental Mg as MgPic; (iv) HFD; rats fed a high-fat diet; (v) HFD + MgO; rats fed a high-fat diet containing 500 mg/kg elemental Mg as MgO; (vi) HFD + MgPic; rats fed a high-fat diet containing 500 mg/kg elemental Mg as MgPic. The Mg sources (MgO (60% elemental Mg) and synthetically produced MgPic (9% elemental Mg, 91% picolinic acid)) were provided by Nutrition 21 LLC. (Harrison, NY, USA). The Mg doses were determined according to Bertinato et al. [30].

At the end of the animal experiment, all rats were sacrificed by cervical dislocation under xylazine (10 mg/kg, i.m.) and ketamine (50 mg/kg, i.m.) anesthesia, and bone tissue samples (the right femur and right tibia) were removed and cleaned of all soft tissues. The samples were stored at −80 °C until analysis.

### 2.3. BMD and Bone Mineral Content Analyses

Mineral density of the tibia and femur was measured by dual-energy X-ray absorptiometry (DXA, Lunar Corp., Madison, WI, USA). Femur Ca, Mg, and Zn levels and diet Mg levels were detected using a microwave digestion system (Speedwave TM MWS-2, Berghoff, Eningen, Germany) and atomic absorption spectroscopy (AAS, PerkinElmer, Analyst 800, Norwalk, USA). Briefly, 300 mg of the femur tissues or diet samples was weighed and transferred into Teflon digestion tubes. The samples were digested with 8 mL nitric acid (65%, *v*/*v*, Suprapur, Merck, Darmstadt, Germany) under a three-step digestion program in a microwave digestion system (Table 2). The mineral levels of the digested samples were measured at 422.7, 285.2, and 213.9 nm wavelength for Ca, Mg, and Zn, respectively, by the flame method in AAS. Each sample was analyzed at least three times. The accuracy of quantitative Ca, Mg, and Zn was assured by simultaneous analysis of certified reference materials (NCS ZC730016 Chicken) which was digested analogously to the samples. The mean recoveries of the reference material for tissues were as follows: 97% Ca, 95% Mg, and 95% Zn.

Bone phosphorus (P) levels were detected by enzymatic colorimetric assay kit (Abcam, ab65622) via microplate reader (Elx-800, Bio-Tek Instruments, Winooski, VT, USA).

### 2.4. Western Blot Analyses

Bone RANK, RANKL, OPG, IGF-1, BMP2, OCN, collagen type 1 alpha 1 (COL1A1), runt-related transcription factor 2 (Runx2), osterix (Osx), and SRY-box transcription factor 9 (SOX9) protein levels were determined through the Western blotting method followed by sodium dodecyl sulfate-polyacrylamide gel electrophoresis (SDS-PAGE). Pooled bone tissues were homogenized with radioimmunoprecipitation assay (RIPA) buffer containing protease and phosphatase inhibitors [31]. Total protein content in the homogenates was measured by Maestro nanodrop spectrophotometer (Maestrogen Inc., Las Vegas, NV, USA). Homogenates containing equal amounts of protein were separated by 12% SDS-PAGE [32]. Proteins were transferred to 0.45 µm pore size nitrocellulose membrane and then blocked by 5% bovine serum albumin to prevent the unspecific bounding. The target primary antibodies RANK, RANKL, OPG, IGF-1, BMP2, OCN, COL1A1, Runx2, Osx, and SOX9 (Santa Cruz Biotechnology, Inc. Dallas, TX, USA) were incubated overnight with a nitrocellulose membrane. Beta-actin antibody (Santa Cruz Biotechnology, Inc. Dallas, Texas, U.S.A.) was used for loading control. Next, nitrocellulose membranes were treated with secondary antibody. Primary-secondary antibody interaction was visualized by staining with chromogenic diaminobenzidine substrate. Finally, membranes scanned and transferred to the Image J program (Version 1.51, National Institutes of Health, Bethesda, Maryland, USA) for densitometric measurements.

### 2.5. Statistical Analysis

The obtained data were analyzed by IBM SPSS (IBM Corp. Released 2012. IBM SPSS Statistics for Windows, Version 22.0. Armonk, NY, USA). The Shapiro–Wilk test or skewness and kurtosis values were used for normality analysis, and the Levene test was used for variance homogeneity evaluation. Normally distributed data were analyzed by one-way ANOVA and Tukey’s or Tamhane’s T2 post hoc test. Non-normal distributed data were analyzed by Kruskal–Wallis and Mann–Whitney U tests with Bonferroni correction (*p* < 0.01). Pearson correlation analysis was performed to determine the relationship between parameters. Data are shown as mean ± standard error of the mean, and *p* < 0.05 indicates a statistical difference.

## 3. Results

### 3.1. BMD

HFD intake resulted in a lower tibia and femur BMD than standard diet intake (*p* < 0.001, Table 3). In control rats, MgO or MgPic supplementation did not affect BMD (*p* > 0.05, Table 3). However, as compared with the HFD group, the tibia BMD levels increased by 7.5% and 14.4% in the HFD + MgO (*p* < 0.01) and HFD + MgPic groups, respectively (*p* < 0.001, Table 3). In addition, femur BMD levels increased by 12.1% in the HFD + MgPic group compared with the HFD group (*p* < 0.001, Table 3).

### 3.2. Bone Ca, P, Mg, and Zn Levels

The bone Ca, P, Mg, and Zn levels were lower in HFD rats as compared with the control rats (*p* < 0.001, Table 4). In rats fed with a standard diet, MgO or MgPic supplementation did not affect bone Ca, P, and Zn levels (*p* > 0.05, Table 4). MgO and MgPic supplementation significantly elevated the bone Mg levels compared with the control group (*p* < 0.001, Table 4). In addition, there was no difference in bone Ca and P levels between the HFD and the HFD + MgO groups (*p* > 0.05, Table 4). MgPic supplementation to the HFD significantly increased bone Ca (*p* < 0.01) and Zn (*p* < 0.001) levels as compared with the HFD group (Table 4). HFD + MgPic group had higher bone P levels as compared with the HFD (*p* < 0.01) and HFD + MgO groups (*p* < 0.05, Table 4). The highest bone Mg levels were detected in the MgO and MgPic groups, while the lowest bone Mg levels were detected in the HFD group (*p* < 0.001, Table 4). As compared with HFD fed rats, dietary MgO and MgPic supplementation elevated bone Mg levels by 26% (*p* < 0.01) and 57% (*p* < 0.001), respectively (Table 4). In addition, bone Zn levels elevated (7.6%) in the HFD + MgO group as compared with the HFD group (*p* < 0.01). The bone Mg level of the HFD + MgPic group was 25% higher than that of the HFD + MgO group (*p* < 0.01, Table 4).

### 3.3. Bone Protein Levels

**Figure 1:**
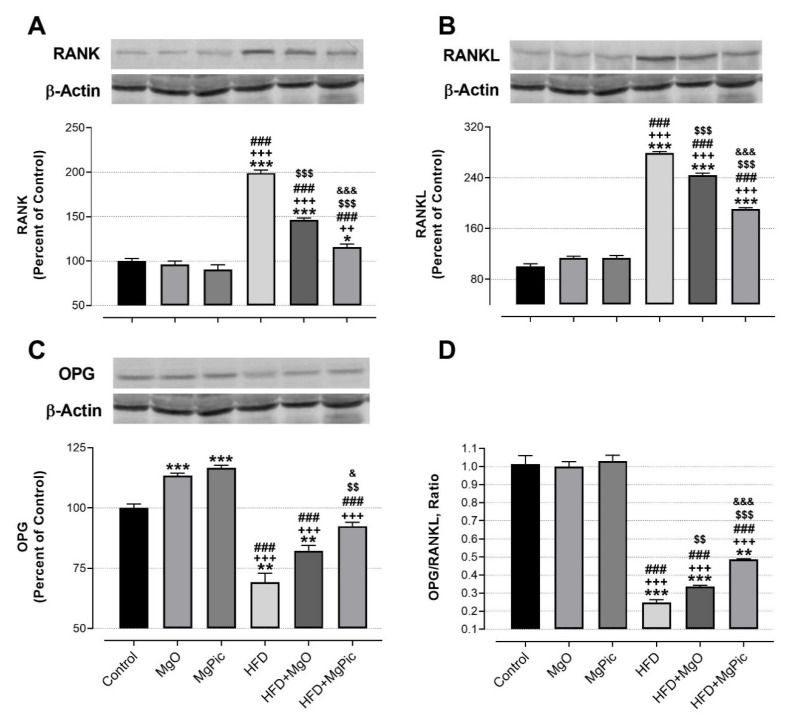
Effects of MgO or MgPic supplementation on bone (**A**) RANK; (**B**) RANKL; (**C**) OPG; (**D**) OPG/RANKL levels in rats. Blots were repeated at least three times, and β-actin was used for loading the control. The blots were cropped, and full-length blots are presented in Supplementary Figure S1. Data (percent of the control group) are shown as mean ± standard error of the mean. Different symbols indicate the statistical difference between groups (* *p* < 0.05, ** *p* < 0.01, and *** *p* < 0.001 as compared with the control group; ^++^ *p* < 0.01 and ^+++^ *p* < 0.001 as compared with the MgO group; ^###^ *p* < 0.001 as compared with the MgPic group; ^$$^ *p* < 0.01 and ^$$$^ *p* < 0.001 as compared with the HFD group; ^&^ *p* < 0.05 and ^&&&^ *p* < 0.001 as compared with the HFD + MgO group). One-way ANOVA and Tamhane’s T2 (OPG and OPG/RANKL) or Tukey’s (RANK and RANKL) post hoc tests were used for statistical analysis.

**Figure 2:**
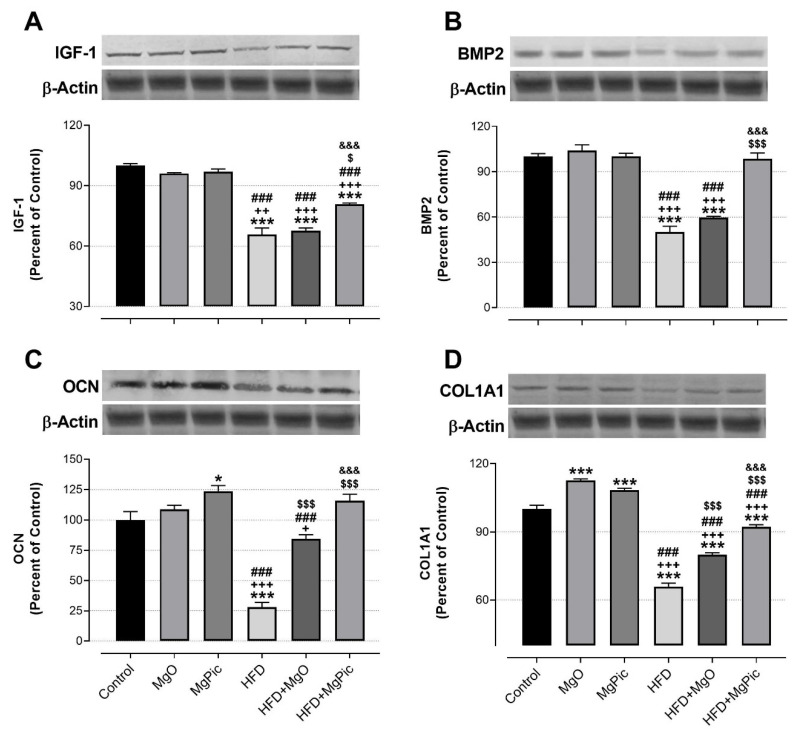
Effects of MgO or MgPic supplementation on bone (**A**) IGF-1; (**B**) BMP2; (**C**) OCN; (**D**) COL1A1 levels in rats. Blots were repeated at least three times, and β-actin was used for loading control. The blots were cropped, and full-length blots are presented in Supplementary Figure S2. Data (percent of the control group) are shown as mean ± standard error of the mean. Different symbols indicate the statistical difference between groups (* *p* < 0.05 and *** *p* < 0.001 as compared with the control group; ^+^ *p* < 0.05, ^++^ *p* < 0.01, and ^+++^ *p* < 0.001 as compared with the MgO group; ^###^ *p* < 0.001 as compared with the MgPic group; ^$^ *p* < 0.05 and ^$$$^ *p* < 0.001 as compared with the HFD group; ^&&&^ *p* < 0.001 as compared with the HFD + MgO group). One-way ANOVA and Tamhane’s T2 (IGF-1) or Tukey’s (BMP2, OCN, and COL1A1) post hoc tests were used for statistical analysis.

**Figure 3:**
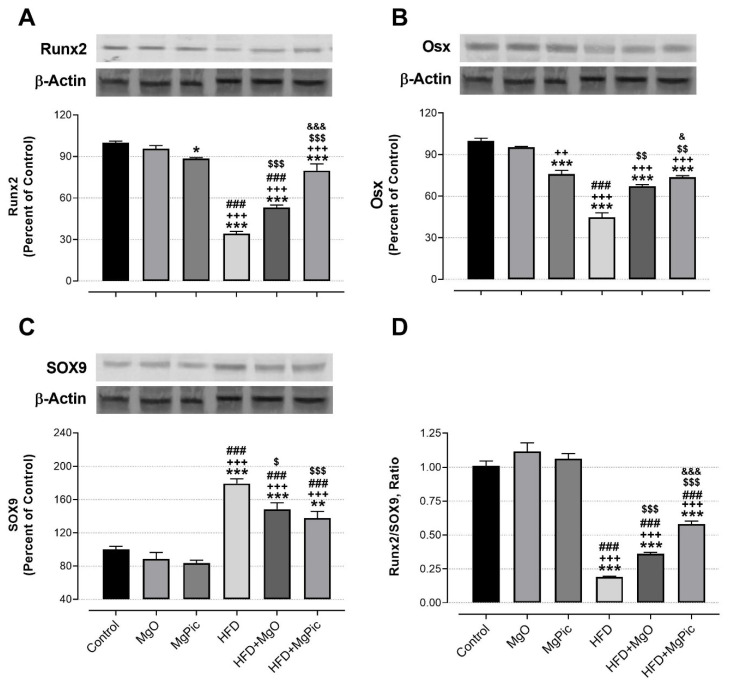
Effects of MgO or MgPic supplementation on bone (**A**) Runx2; (**B**) Osx; (**C**), SOX9; (**D**) Runx2/SOX9 levels in rats. Blots were repeated at least three times, and β-actin was used for loading control. The blots were cropped, and full-length blots are presented in Supplementary Figure S3. Data (percent of the control group) are shown as mean ± standard error of the mean. Different symbols indicate the statistical difference between groups (* *p* < 0.05, ** *p* < 0.01, and *** *p* < 0.001 as compared with the control group; ^++^ *p* < 0.01 and ^+++^ *p* < 0.001 as compared with the MgO group; ^###^ *p* < 0.001 as compared with the MgPic group; ^$^ *p* < 0.05, ^$$^ *p* < 0.01, and ^$$$^ *p* < 0.001 as compared with the HFD group; ^&^ *p* < 0.05 and ^&&&^ *p* < 0.001 as compared with the HFD + MgO group). One-way ANOVA and Tamhane’s T2 (Osx) or Tukey’s (Runx2 and SOX9) post hoc test were used and for statistical analysis. Kruskal–Wallis and Mann–Whitney U tests with Bonferroni correction were used for the Runx2/SOX9 ratio (*p* < 0.01).

HFD intake significantly reduced the femur OPG (Figure 1C, *p* < 0.01), IGF-1, BMP2, OCN, COL1A1 (Figure 2, *p* < 0.001), Runx2 (Figure 3A, *p* < 0.001), and Osx (Figure 3B, *p* < 0.001) protein levels, whereas it increased the RANK (Figure 1A), RANKL (Figure 1B), and SOX9 (Figure 3C) levels (*p* < 0.001, for all). In addition, the OPG/RANKL (Figure 1D) and Runx2/SOX9 (Figure 3D) ratios were reduced by HFD intake (*p* < 0.001). MgO supplementation to the HFD decreased the bone RANK (Figure 1A, *p* < 0.001), RANKL (Figure 1B, *p* < 0.001), and SOX9 (Figure 3C, *p* < 0.05) protein levels by 26.5%, 12.5%, and 17.1%, respectively. On the contrary, as compared with the HFD group, the bone OCN (Figure 2C, *p* < 0.001), COL1A1 (Figure 2D, *p* < 0.001), Runx2 (Figure 3A, *p* < 0.001), and Osx (Figure 3B, *p* < 0.01) levels increased in the HFD + MgO group by 302%, 21.4%, 55.6%, and 50%, respectively. In addition, the OPG/RANKL (*p* < 0.01) and Runx2/SOX9 (*p* < 0.001) ratios increased in the HFD + MgO group as compared with the HFD group. Likewise, as compared with the HFD group, MgPic supplementation to the HFD reduced the bone RANK (21%), RANKL (31.8%), and SOX9 (23.1%) levels (*p* < 0.001, for all), whereas it increased the bone OPG (33.5%, *p* < 0.01), IGF-1 (23% *p* < 0.05), BMP2 (96.7%, *p* < 0.001), OCN (454% *p* < 0.001), COL1A1 (34%, *p* < 0.001), Runx2 (133.1%, *p* < 0.001), and Osx (64.6%, *p* < 0.01) levels. The OPG/RANKL (*p* < 0.01) and Runx2/SOX9 (*p* < 0.001) ratios increased in the HFD + MgPic group as compared with the HFD group (*p* < 0.001, for all). In addition, as compared with the HFD + MgO group, the RANK and RANKL proteins decreased in the HFD + MgPic group (*p* < 0.001), whereas the bone OPG (*p* < 0.05), IGF-1, OCN, BMP2, COL1A1, Runx2 (*p* < 0.001 for all), and Osx (*p* < 0.05) levels increased. In addition, MgPic supplementation elevated the OPG/RANKL, and Runx2/SOX9 ratios in HFD fed rats as compared with MgO supplementation (*p* < 0.001).

### 3.4. The Correlation between BMD, Ca, P, Mg, Zn, and Target Protein Levels

There was a positive correlation between tibia BMD, femur BMD, bone Ca, P, Mg, Zn, bone OCN, IGF-1, OPG, COL1A1, BMP2, Runx2, and Osx protein levels (*p* < 0.01, for all, Table 5). On the contrary, a negative correlation was observed between osteogenic parameters and bone RANK, RANKL, and SOX9 protein levels (*p* < 0.01, for all, Table 5).

## 4. Discussion

Previous studies have shown that HFD and Western diet can reduce BMD levels [19,33,34,35,36]. Feeding with HFD may interfere with intestinal mineral absorption and renal reabsorption [22], especially Mg and Ca [21,37]. This may have decreased bone Ca, P, Mg, and Zn levels in the HFD rats. Similarly, Kang et al. [35] reported that femur BMD and tibia BMD levels decreased after eight weeks in HFD fed rats [35].

Mineral supplements can be beneficial in preventing or treating bone diseases with bone loss [38,39]. For example, Liu et al. [40] showed that Mg supplementation prevented bone resorption and increased bone formation in growing male rats [40]. Similarly, a meta-analysis study reported increased BMD (r 0.16, 95% CI 0.001) in humans associated with Mg consumption [41]. Similar to Karaaslan et al. [42], our results demonstrate a correlation between BMD increased after Mg supplementation and bone Mg, Ca, P, and Zn levels [42].

HFD intake triggers proinflammatory cytokines and increases osteoclastic activity in bone [43]. It has been shown that the increasing osteoclastic activity mainly depends on elevated RANK/RANKL interaction stimulated by TNF-α in rats [44]. Xiao et al. [18] reported that consumption of HFD decreased the bone OPG/RANKL ratio and IGF-1 level while increasing the bone RANKL level by producing inflammatory cytokines such as IL-1, IL-6, and TNF-α in mice [18]. In addition, excessive production of these inflammatory cytokines may intercept BMP2-related bone mass [45]. BMP2 stimulates Runx2 and Osx that are required for osteoblastic differentiation of mesenchymal stem cells. Subsequently, Runx2 and Osx activate transcription factors of OCN and COL1A1 [46]. Consistent with our results, Adhikary et al. [20] demonstrated that bone BMP2, Osx, OCN, COL1A1, and OPG levels were reduced in HFD fed mice [20]. Moreover, we detected that the bone OPG, IGF-1, Runx2, Osx, OCN, and COL1A1 levels were negatively correlated to bone RANK and RANKL proteins.

Mg could reverse the RANKL-mediated osteoclastic activity due to its anti-inflammatory function and prevent osteocyte apoptosis [8]. Mg supplementations may suppress the RANK/RANKL pathway [47] and stimulate OPG signaling in the osteoblast [48]. In addition, Mg has IGF-1-related anabolic effects on bone by initiating the IGF-1/PI3K/Akt cascade [8,49]. Similar to the previous studies, we found that Mg supplementations increased bone BMP2 [50], BMP2-related Runx2, Osx, OCN [51], and type 1 collagen levels [10]. Furthermore, Mg probably induced osteoblastic proliferation through the Wnt/β-catenin, Notch [51], and Smad [49] signaling pathways. The positive correlation between bone Mg level and osteogenic proteins also supports the consistency of our results. Thus, it can be concluded that Mg maintains osteoblastic activity to balance bone homeostasis.

Runx2 and SOX9 regulate each other to transform bone mesenchymal stem cells to the preosteoblasts or chondrocytes. Runx2 enhances endochondral bone formation by suppressing SOX9 expression. In contrast, when Runx2 is insufficient, SOX9 stimulates chondrogenesis [52,53]. Although SOX9 is decreased in cartilage under inflammatory conditions, we found that bone SOX9 levels increased after HFD intake. This result may be due to reduced bone Runx2 and Osx levels in HFD fed rats. Therefore, in this study, theRunx2/SOX9 ratio was also reduced in the HFD group. It has been reported that Mg^+2^ ions decreased Runx2 expression while it increased SOX9 expression in chondrocytes [54]. Conversely, Li, et al. [55] demonstrated that different Mg alloys increased the TGFβ, Smad4, BMP2, and COL1A1 expressions, whereas they decreased SOX2 and SOX9 expressions in bone marrow mesenchymal stem cells [55]. In addition, we demonstrated that there was a negative correlation between bone SOX9 and Runx2. However, no in vivo study was found comparing our results. Therefore, for the first time, we show that dietary Mg has a regulatory effect on SOX9 protein in bone in vivo.

This study showed that MgPic effectively elevated the bone Ca, P, Mg, and Zn levels as compared with MgO. In addition, there was no statistical difference between the control and the HFD + MgPic groups in terms of bone Ca and Mg levels. In addition, MgPic increased bone OPG, IGF-1, OCN, BMP2, COL1A1, Runx2, and Osx protein levels compared with MgO in HFD fed rats. However, no studies were found comparing the effects of MgPic on bone metabolism.

Picolinic acid, which has a highly chelating capacity, improves Mg bioavailability in synthetic MgPic form [25]. Recently, Orhan et al. [26] reported that MgPic increased Mg bioavailability as well as serum and liver Mg levels more than MgO in rats [26]. Similarly, our study showed that BMD levels increased in parallel with bone Mg levels in rats after MgPic supplementation. Furthermore, Orhan et al. [27] found that MgPic supplementation was more effective than MgO in preventing HFD-induced oxidative retinal damage in rats [27]. The bone RANK level decreased by MgPic supplementation in HFD rats. Thus, the OPG/RANKL ratio increased more effectively with MgPic supplementation. In addition, picolinic acid, which has a BMD increasing effect [56], could increase bone Runx2 and OCN levels via stimulating Wnt/β-catenin signaling pathway similar to Mg [56,57]. These common properties of Mg and picolinic acid may have contributed to the effect of MgPic on preventing bone loss.

In conclusion, we report that organic MgPic can alleviate HFD induced bone metabolism disorders in an animal model for the first time. This effect most likely stems from the higher bioavailability of MgPic than MgO. MgPic consumption could increase Mg accumulation in bone tissue, as well as soft tissues. Due to this efficacy, MgPic showed higher osteogenic and anti-osteoclastogenic activity than MgO in HFD fed rats. Firstly, MgPic decreased bone RANK and RANKL levels and increased decoy receptor OPG levels. These results indicate that MgPic might have regulated the RANK/RANKL/OPG pathway. Additionally, MgPic triggered IGF-1 and BMP2 dependent transcription factors that provide osteoblast proliferation, such as Runx2 and Osx. Finally, MgPic increased the production of OCN and COL1A1, which are the main bone proteins.

In addition, these results suggest that in obese, overweight, and diabetic individuals, MgPic supplements may alleviate clinical bone disorders such as osteoporosis. Furthermore, postmenopausal women could prefer to intake the organic Mg forms instead of inorganic ones to prevent osteoporosis. We propose that this study could shed light on future studies on the effects of Mg supplements such as MgPic on disorders in bone metabolism caused by malnutrition or metabolic diseases. However, clinical studies on the effects of Mg forms on bone metabolism are needed.

## Figures and Tables

**Table 1:** Composition of experimental diets *.

	Control Diet	High-Fat Diet
Ingredients, %		
Casein	20.00	20.00
Cornstarch	57.95	15.00
Sucrose	5.00	14.95
Soy oil	7.00	-
Beef tallow	-	40.00
Cellulose	5.00	5.00
Vitamin-mineral premix **	4.50	4.50
L- cysteine	0.30	0.30
Choline bitartrate	0.25	0.25
Analyses		
Crude protein, %	17.90	17.90
Eter extract, %	6.90	39.90
Crude fiber, %	5.00	5.00
Ash, %	4.2	4.2
Metabolic energy, kcal/kg	3820	4810

* Control and HFD diets contain 478.80 ± 45.20 and 454.50 ± 60.70 mg elemental Mg per kg diet, respectively. ** Vitamin-mineral premix (g/kg, AIN-93G-MX/AIN-93-VX (2/1)0: calcium carbonate (CaCO_3_) 238, monobasic potassium phosphate (KH_2_PO_4_) 130.7, sodium chloride (NaCl) 49, potassium sulfate (K₂SO₄) 31.01, tri-potassium citrate monohydrate (C_6_H_5_K_3_O_7_*H_2_O) 41.19, ferric citrate (C_6_H_5_FeO_7_) 4.04, zinc carbonate (ZnCO₃) 1.1, manganese carbonate (MnCO_3_) 0.42, copper carbonate (CuCO_3_) 0.2, potassium iodate (KIO) 0.0067, sodium selenite (Na_2_SeO_3_) 0.0068, ammonium paramolybdate tetrahydrate (H_32_Mo_7_N_6_O_28_) 0.0053, niacin (Vitamin B3) 1, calcium pantothenate (Vitamin B5) 0.53, pyridoxine-HCl (Vitamin B6) 0.23, thiamine HCl (Vitamin B1) riboflavin (Vitamin B2) 0.07, folic acid (Vitamin B9) 0.07, d-biotin (Vitamin B7) 0.007, Vitamin B12 (0.1% cyanocobalamin) 0.83, Vitamin E (all-rac-α-tocopheryl acetate, 500 IU/g) 5, Vitamin A (all-trans-retinyl palmitate, 500,000 IU/g) 0.27, Vitamin D3 (cholecalciferol, 400,000 IU/g) 0.083, Vitamin K (phylloquinone) 0.025.

**Table 2:** Three-step microwave digestion program.

	Step 1			Step 2			Step 3		
	°C	Time (min)	Power (%)	°C	Time (min)	Power (%)	°C	Time (min)	Power (%)
Bone Tissue	145	10	80	160	10	80	190	20	80
Diet Samples	130	8	80	155	5	80	170	12	80

**Table 3:** Effect of MgO or MgPic supplementation on BMD in rats.

	Tibia BMD, mg/cm^2^	Femur BMD, mg/cm^2^
Control	222.18 ± 3.69	234.68 ± 3.96
MgO	225.83 ± 3.26	230.51 ± 3.52
MgPic	226.01 ± 2.38	231.6 ± 1.93
HFD	171.86 ± 3.43 ***^, +++,###^	186.88 ± 3.32 ***^,+++,###^
HFD + MgO	184.72 ± 1.48 ***^,+++,###,$$^	196.92 ± 2.95 ***^,+++,###^
HFD + MgPic	196.65 ± 2.47 ***^,+++,###,$$$^	209.46 ± 2.94 ***^,+++,###,$$$^
	*p* < 0.001	*p* < 0.001

Data are shown as mean ± standard error of the mean. Upper symbols in the same column indicate the statistical difference between groups (*** *p* < 0.001 as compared with the control group; ^+++^ *p* < 0.001 as compared with the MgO group; ^###^ *p* < 0.001 as compared with the MgPic group; ^$$^ *p* < 0.01, ^$$$^ *p* < 0.001 as compared with the HFD group). One-way ANOVA, Tukey’s post hoc test.

**Table 4:** Effects of MgO or MgPic supplementation on bone Ca, P, Mg, and Zn levels in rats.

	Ca (mg/g)	P (mg/g)	Mg (mg/g)	Zn (mg/g)
Control	191.61 ± 2.75	141.61 ± 1.91	4.53 ± 0.13	230.60 ± 2.55
MgO	193.46 ± 1.64	142.27 ± 1.76	6.28 ± 0.19 ***	226.62 ± 5.95
MgPic	194.51 ± 2.73	143.37 ± 1.87	7.1 ± 0.18 ***	272.70 ± 4.01
HFD	170.88 ± 2.84 ***^, +++, ###^	118.37 ± 1.96 ***^, +++, ###^	3.26 ± 0.07 ***^, +++, ###^	176.23 ± 1.60 ***^, ++^
HFD + MgO	177.91 ± 2.02 ***^, ++, ###^	122.57 ± 2.69 ***^, +++, ###^	4.11 ± 2.75 ^+++, ###, $$^	189.53 ± 2.22 ***^, ++, $$^
HFD + MgPic	184.46 ± 1.83 ^#, $$^	132.06 ± 2.75 *^, +, #, $$, &^	5.13 ± 0.13 ^++, ###, $$$, &&^	195.90 ± 2.07 ***^, +, $$$^
	*p* < 0.001	*p <* 0.001	*p <* 0.001	*p* < 0.001

Data are shown as mean ± standard error of the mean. Upper symbols in the same column indicate the statistical difference between groups (* *p* < 0.05 and *** *p* < 0.001 as compared with the control group; ^+^ *p* < 0.05, ^++^ *p* < 0.01, and ^+++^ *p* < 0.001 as compared with the MgO group; ^#^ *p* < 0.05 and ^###^ *p*< 0.001 as compared with the MgPic group; ^$$^ *p* < 0.01 and ^$$$^ *p* < 0.001 as compared with the HFD group; ^&^ *p* < 0.05 and ^&&^ *p* < 0.01 as compared with the HFD + MgO group). One-way ANOVA and Tamhane’s T2 (Mg and Zn) or Tukey’s (Ca and P) post hoc test.

**Table 5:** The correlation between BMD, bone Ca, P, Mg, Zn, and target protein levels.

	Femur BMD	Ca	P	Mg	Zn	Rank	Rankl	OPG	IGF−1	BMP2	OCN	COL1A1	Runx2	Osx	SOX9
Tibia BMD	0.893	0.776	0.824	0.768	0.899	−0.864	−0.930	0.876	0.883	0.800	0.703	0.917	0.868	0.774	−0.863
Femur BMD		0.745	0.847	0.687	0.859	−0.839	−0.925	0.834	0.866	0.772	0.702	0.873	0.847	0.801	−0.828
Ca			0.743	0.730	0.747	−0.810	−0.830	0.799	0.771	0.759	0.609	0.821	0.797	0.672	−0.737
P				0.718	0.826	−0.798	−0.861	0.779	0.817	0.741	0.655	0.813	0.788	0.712	−0.834
Mg					0.724	−0.807	−0.739	0.870	0.693	0.710	0.740	0.855	0.689	0.489	−0.756
Zn						−0.804	−0.909	0.840	0.878	0.716	0.661	0.857	0.856	0.760	−0.816
RANK							0.908	−0.868	−0.860	−0.875	−0.863	−0.919	−0.924	−0.831	0.821
RANKL								−0.879	−0.948	−0.872	−0.719	−0.931	−0.936	−0.870	0.877
OPG									0.821	0.834	0.795	0.958	0.852	0.707	−0.796
IGF−1										0.822	0.653	0.863	0.892	0.832	−0.822
BMP2											0.779	0.897	0.904	0.763	−0.751
OCN												0.810	0.767	0.682	−0.690
COL1A1													0.905	0.793	−0.865
Runx2														0.894	−0.740
Osx															−0.700

The values show the Pearson correlation coefficients (r, *p* < 0.01, for all).

## Data Availability

Data are contained within this article.

## Supplementary Materials

The following are available online at https://www.mdpi.com/article/10.3390/nu13103353/s1.
