# Supplementary material for: Magnesium Picolinate Improves Bone Formation by Regulation of RANK/RANKL/OPG and BMP-2/Runx2 Signaling Pathways in High-Fat Fed Rats
Source: Nutrients. 2021 Sep 24;13(10):3353. doi: 10.3390/nu13103353 (PMC8538721; doi:10.3390/nu13103353)
Supplement: Supplementary file 1 [file nutrients-13-03353-s001.zip › nutrients-1391989-Supplementary.pdf]

Magnesium picolinate improves bone formation by regulation of RANK/RANKL/OPG and BMP-2/Runx2 signaling pathways in high-fat-fed rats

Emre Sahin[1], Cemal Orhan[1] Tansel Ansal Balci[2] Fusun Erten[3], Kazim Sahin[1*],

[1]Department of Animal Nutrition, Faculty of Veterinary Medicine, Firat University, Elazig, Turkey; esahin@bingol.edu.tr (E.S.); nsahinkm@yahoo.com (K.S.); corhan@firat.edu.tr (C.O.)

[2]Department of Nuclear Medicine, School of Medicine, Firat University, Elazig, Turkey ; tanselbalci@firat.edu.tr (T.A.B.)

[3]Department of Veterinary Medicine, Pertek Sakine Genc Vocational School, Munzur University, Tunceli, Turkey; fusunerten@munzur.edu.tr (F.E.)

*****Correspondence: nsahinkm@yahoo.com (K.S.); Tel.: +90-532-7473506 Phone: +904242370000/3938

(a) RANK

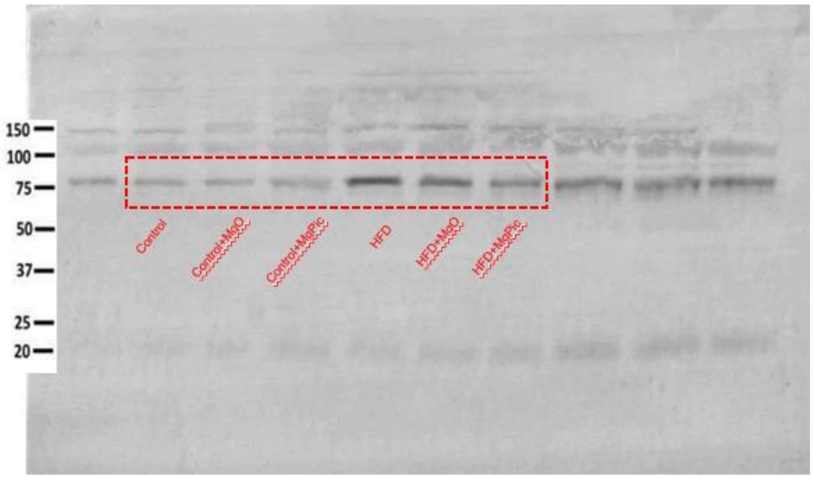

(b) RANKL

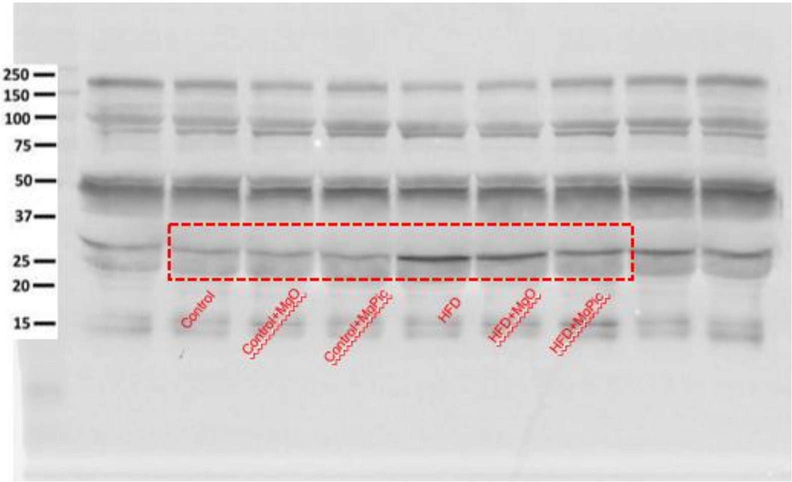

(c) OPG

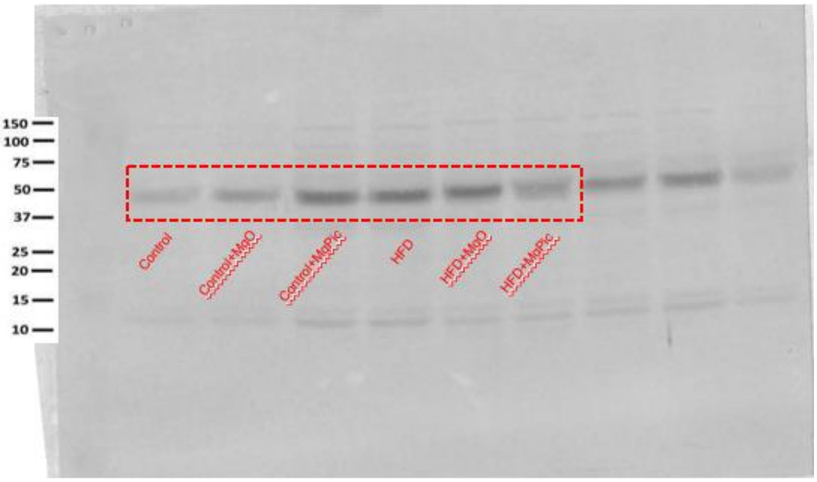

(d) β-Actin

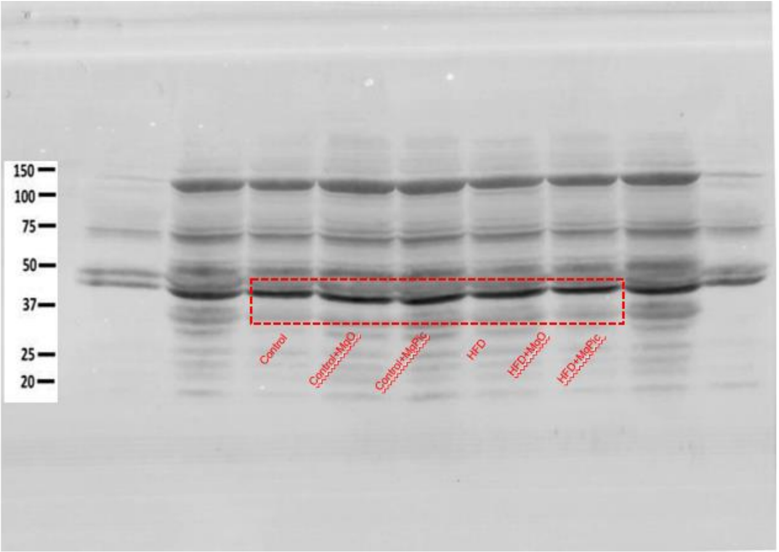

Figure. S1. Full immunoblots related to Fig. 1 on bone tissue in rats ((**a**) RANK, (**b**) RANKL, (**c**) OPG, and (**d**) β-Actin)). Each immunoblot is a representative of three independent experiments. Results shown in Fig.1 are delineated by red dotted rectangles. MW (in kDa) are indicated.

(a) IGF-1

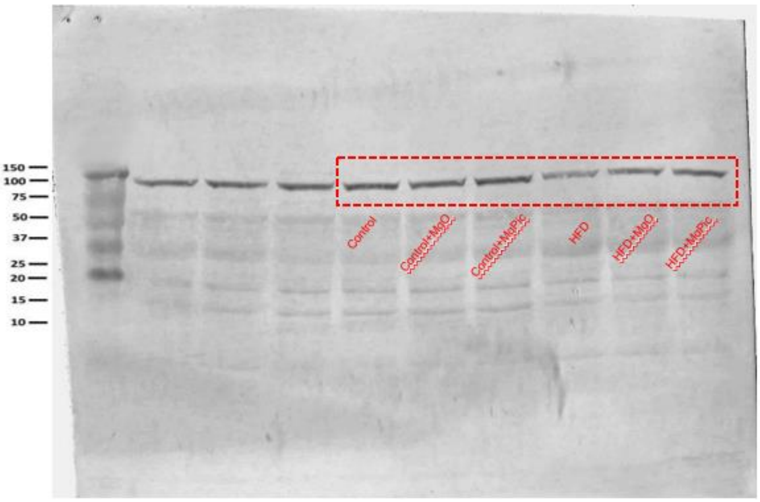

(b) BMP2

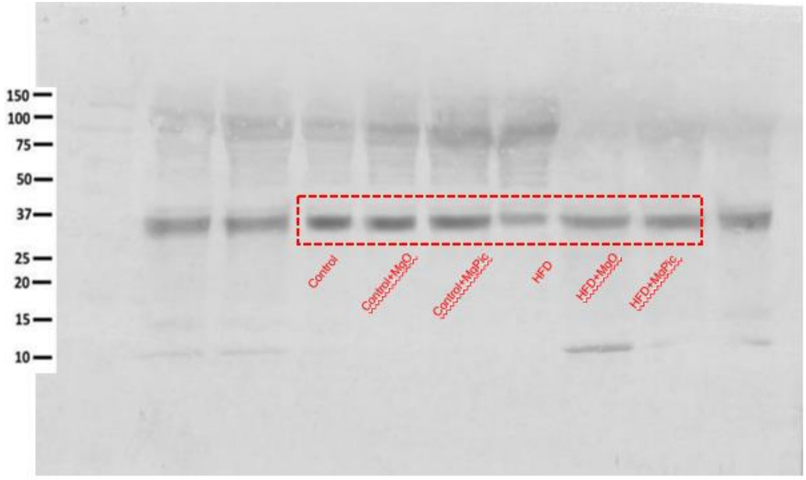

(c) OCN

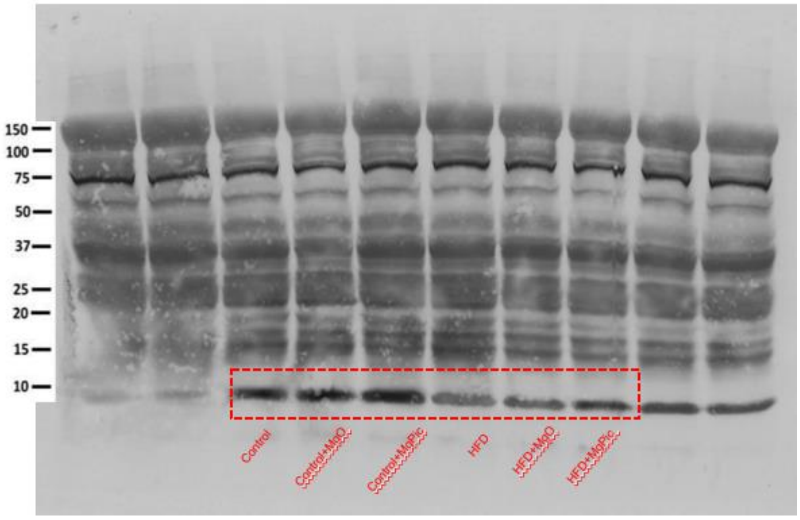

(d) COL1A1

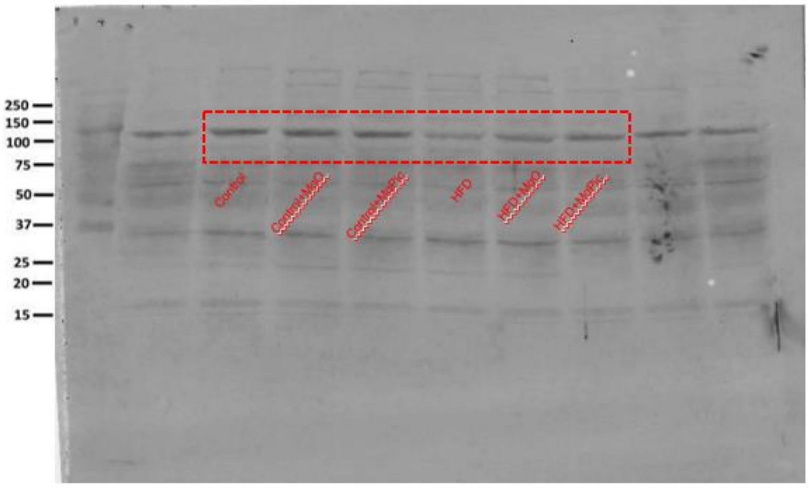

(e) β-Actin

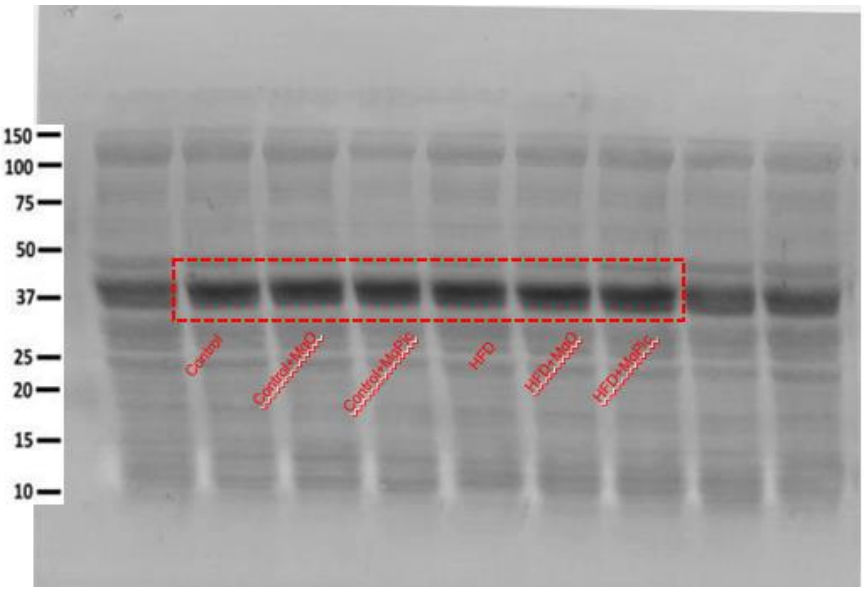

Figure. S2. Full immunoblots related to Fig. 2 on bone tissue in rats ((**a**) IGF-1, (**b**) BMP2, (**c**) OCN, (**d**) COL1A1, and (**e**) β-Actin)). Each immunoblot is a representative of three independent experiments. Results shown in Fig.2 are delineated by red dotted rectangles. MW (in kDa) are indicated.

(a) Runx2

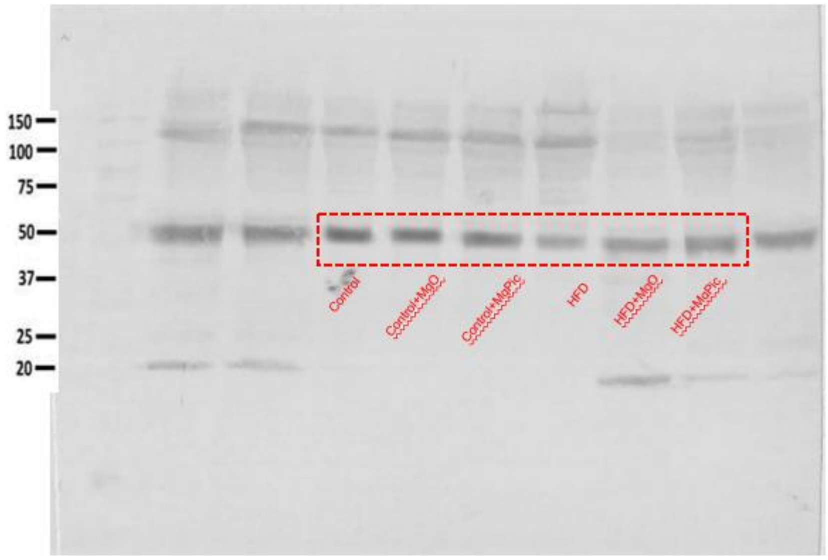

(b) Osx

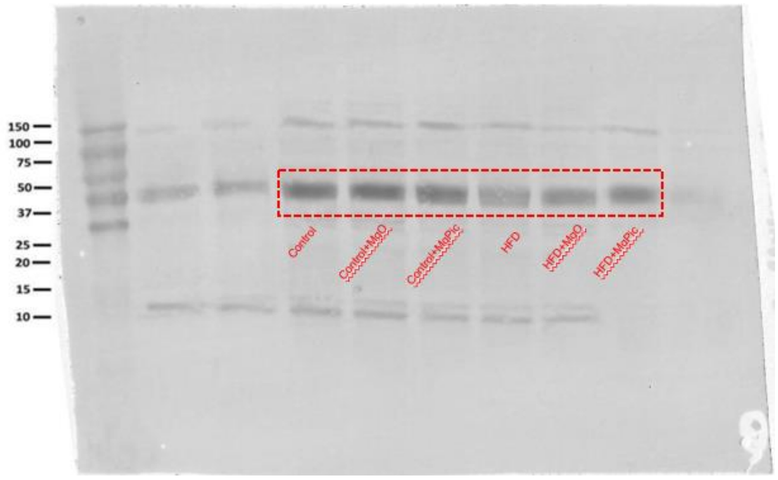

(c) SOX9

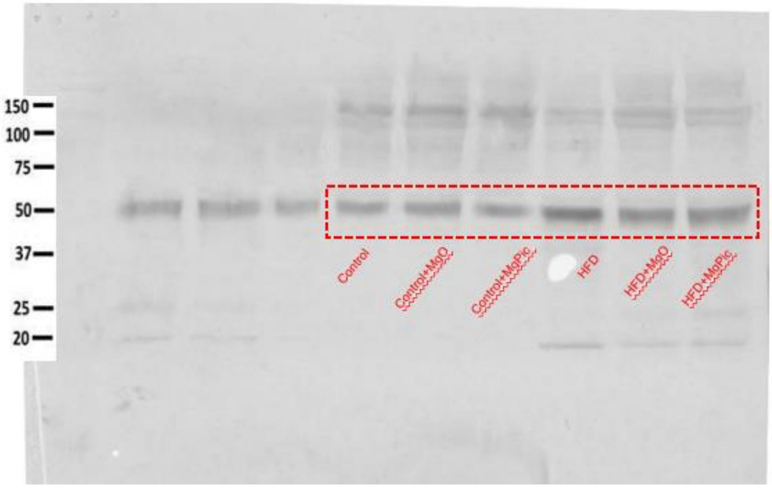

(d) β-Actin

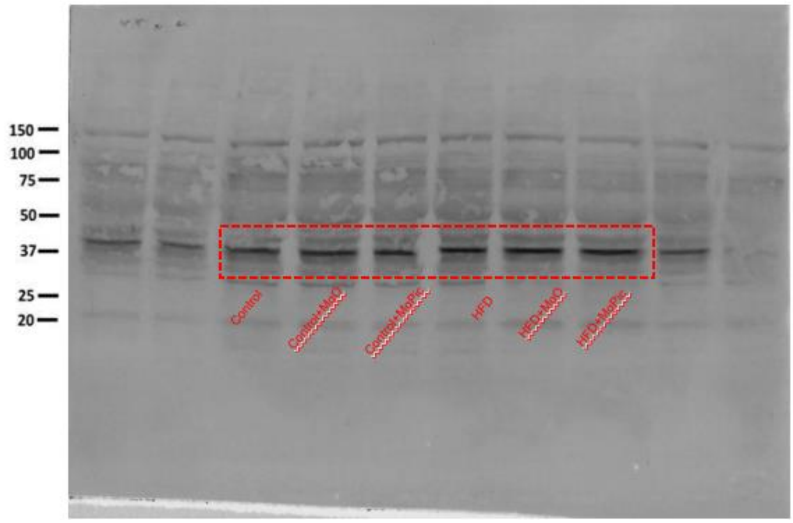

Figure. S3. Full immunoblots related to Fig. 3 on bone tissue in rats ((**a**) Runx2, (**b**) Osx, (**c**) SOX9, and (**d**) β-Actin)). Each immunoblot is a representative of three independent experiments. Results shown in Fig.3 are delineated by red dotted rectangles. MW (in kDa) are indicated.
